# Colonized *Sabethes cyaneus*, a Sylvatic New World Mosquito Species, Shows a Low Vector Competence for Zika Virus Relative to *Aedes aegypti*

**DOI:** 10.3390/v10080434

**Published:** 2018-08-16

**Authors:** Ajit K. Karna, Sasha R. Azar, Jessica A. Plante, Rumei Yun, Nikos Vasilakis, Scott C. Weaver, Immo A. Hansen, Kathryn A. Hanley

**Affiliations:** 1 Department of Biology, New Mexico State University, Las Cruces, NM 88003, USA; akarna@nmsu.edu (A.K.K.); immoh@nmsu.edu (I.A.H.); 2 Department of Microbiology and Immunology, University of Texas Medical Branch, Galveston, TX 77555, USA; srazar@utmb.edu (S.R.A.); japlante@utmb.edu (J.A.P.); ruyun@utmb.edu (R.Y.); sweaver@utmb.edu (S.C.W.); 3 Department of Pathology, University of Texas Medical Branch, Galveston, TX 77555, USA; nivasila@utmb.edu; 4 Institute for Human Infections and Immunity, University of Texas Medical Branch, Galveston, TX 77555, USA; 5 Center for Biodefense and Emerging Infectious Diseases, University of Texas Medical Branch, Galveston, TX 77555, USA; 6 Institute for Translational Sciences, University of Texas Medical Branch, Galveston, TX 77555, USA; 7 Center for Tropical Diseases, University of Texas Medical Branch, Galveston, TX 77555, USA

**Keywords:** *Sabethes cyaneus*, Zika virus, *Aedes aegypti*, sylvatic cycle, vector competence, Americas, spillback

## Abstract

The introduction of Zika virus (ZIKV) to the Americas raised concern that the virus would spill back from human transmission, perpetuated by *Aedes aegypti*, into a sylvatic cycle maintained in wildlife and forest-living mosquitoes. In the Americas, *Sabethes* species are vectors of sylvatic yellow fever virus (YFV) and are therefore candidate vectors of a sylvatic ZIKV cycle. To test the potential of *Sabethes cyaneus* to transmit ZIKV, *Sa. cyaneus* and *Ae. aegypti* were fed on A129 mice one or two days post-infection (dpi) with a ZIKV isolate from Mexico. *Sa. cyaneus* were sampled at 3, 4, 5, 7, 14, and 21 days post-feeding (dpf) and *Ae. aegypti* were sampled at 14 and 21 dpf. ZIKV was quantified in mosquito bodies, legs, and saliva to measure infection, dissemination, and potential transmission, respectively. Of 69 *Sa. cyaneus* that fed, ZIKV was detected in only one, in all body compartments, at 21 dpf. In contrast, at 14 dpf 100% of 20 *Ae. aegypti* that fed on mice at 2 dpi were infected and 70% had virus in saliva. These data demonstrate that *Sa. cyaneus* is a competent vector for ZIKV, albeit much less competent than *Ae. aegypti*.

## 1. Introduction

Mosquito-borne Zika virus (ZIKV; genus *Flavivirus*) originated in a sylvatic cycle of transmission between arboreal *Aedes* mosquitoes and wildlife, including non-human primates, in Africa [1,2]. Between its discovery in 1947 and 2006, the virus was detected in a limited range within the tropical belt of Africa and Asia, and human cases during this time were primarily attributable to spillover from the sylvatic cycle [1]. However, in 2007, an epidemic of ZIKV occurred on Yap island, where the virus was most likely transmitted by *Ae.* (*Stegomyia*) *hensilii* [3]. In 2013, ZIKV invaded French Polynesia and then the Americas, where it initiated explosive spread among humans via the urban vector *Ae.* (*Stegomyia*) *aegypti* [1,2,4]. At this time, the link between Zika virus infection of pregnant women and Zika congenital syndrome, particularly the manifestation of microcephaly, was first established [5].

The introduction of ZIKV to the New World raised concern that the virus would spill back into a sylvatic cycle in its new range [1,6]. We recently demonstrated, via mathematical modeling, that ZIKV does have high potential for such spillback in Brazil [6]. A sylvatic ZIKV cycle would preclude eradication of the virus from the Americas via human vaccination and undermine ZIKV control efforts [1,6]. The spillback of YFV, another flavivirus, which was introduced into the New World centuries ago via the slave trade, illustrates the potential for flaviviruses to establish novel sylvatic cycles and the risks such cycles pose [7,8]. YFV circulates between non-human primates and canopy-living mosquitoes, particularly *Haemagogus* and *Sabethes*, in the Americas [8]. Spillover from this cycle is currently driving an unprecedented outbreak of yellow fever among humans in Brazil [9].

One of the caveats of our modeling study was that more data was urgently needed on susceptibility of New World primates and sylvatic mosquitoes to ZIKV [6]. Subsequently, we and others have demonstrated that New World monkeys are susceptible to ZIKV in the laboratory [10,11,12] and that free-living monkeys are becoming infected with ZIKV in Brazil [9,13]. However, to our knowledge, the vector competence of sylvatic New World mosquitoes for ZIKV has not yet been tested. To help close this knowledge gap, we tested the vector competence of *Sabethes cyaneus*, which inhabits neotropical forests from Belize south to Argentina (http://www.mosquitocatalog.org/taxon_descr.aspx?ID=15874) for ZIKV.

## 2. Materials and Methods

### 2.1. Virus Strains and Cell Lines

*Ae. albopictus* (C6/36) cells were maintained in minimum essential medium (MEM) supplemented with 10% heat-inactivated fetal bovine serum (FBS), 2 mM l-glutamine, 2 mM non-essential amino acids (all from Gibco, Life Technologies, Grand Island, NY, USA), and 0.05 mg/mL gentamycin (Invitrogen, Life Technologies, Grand Island, NY, USA) at 32 °C, 5% CO_2_, and 85% relative humidity (RH). African green monkey kidney (Vero) cells (CCL-81) were maintained in DMEM supplemented with 5% FBS and Penicillin/Streptomycin (P/S; 100 Units/mL and 100 μg/mL respectively) at 37 °C, 5% CO_2_, and 85% RH. Zika virus (strain Mex-1-7 obtained from the University of Texas Medical Branch’s World Reference Center for Emerging Viruses and Arboviruses) was used to infect mosquitoes and mice. ZIKV MEX 1-7 (Genbank accession. No. KX247632) was originally isolated from *Ae*, *aegypti* from Chiapas (Mexico) in 2015, and subsequently passaged on Vero (4x), C6/36 (1x), and Vero (3x) and C6/36 (2x) cells.

### 2.2. Mosquito Strains

Although the particular species *Sa. cyaneus* is not a known vector of arboviruses, it was chosen for this study because there is a stable colony available, and at least a subset of the individuals of this colony are willing to feed on mice. The Majé strain of *Sa. cyaneus* was isolated by Dr. Woodbridge Foster on Isla de Majé, Lago Bayano in eastern Panama in 1988 and maintained continuously at The Ohio State University until 2016, when a portion of the colony was transferred to New Mexico State University. *Sa. cyaneus* were maintained at 28 °C and 80% RH in BugDorm cages (30 × 30 × 30 cm, Bioquip, Rancho Dominguez, CA, USA) provisioned with branches that served as mating locations, artificial tree holes containing water for oviposition, and two separate Erlenmeyer flasks with a cotton ball wick, one each containing water or 20% sucrose. The mosquitoes were allowed to feed weekly on one investigator’s (I.A. Hansen) hand for 15–30 min for blood meal (per approved NMSU IRB application 15308). Eggs were transferred from the artificial tree holes into rearing. Larvae were fed with dry cat food pellets (Special Kitty, Walmart, Bentonville, AR, USA) *ad lib*. See Supplementary Video 1 for a detailed protocol for maintenance of *Sa. cyaneus*. An *Ae. aegypti* strain from Salvador, Brazil at the F7 generation was used as a benchmark for vector competence; these were maintained as previously described [14]. Both species were reared in a common incubator at the University of Texas Medical Branch for the infection studies at 27 °C ± 1 °C, 16:8 L:D, 80% RH and maintained in this incubator after feeding on infected mice.

### 2.3. Mouse Infections and Mosquito Feeding

We have previously shown that ZIKV is more infectious to mosquitoes that imbibe the virus from a live, viremic host than from an artificial bloodmeal [14]. Thus we infected A129 mice lacking the interferon (IFN) alpha and beta-receptor with ZIKV as described in detail previously [14]. Briefly, three-week-old mice were injected intraperitoneally with 1 × 10^5^ pfu/mouse ZIKV. To facilitate mosquito feeding, mice were anesthetized via intraperitoneal injection with 100 mg/kg ketamine and 10 mg/kg xylazine in a volume of 100 µL Cartons of *Sa. cyaneus* or *Ae. aegypti* were allowed to feed on mice one day or two dpi for 30 min, after which they were cold-anesthetized and engorged mosquitoes were separated and returned to normal maintenance conditions. Serum was collected from each infected mouse after mosquito feeding and the virus titer was determined using a focus-forming assay as previously described [14]. The titers of these samples were 5.38 log_10_ pfu/mL at 1 dpi and 6.83 log_10_ pfu/mL at 2 dpi, respectively. All animal procedures and manipulations were approved by the UTMB Institutional Animal Care and Use Committee (IACUC) (protocol #170851 approved 2 February 2018).

### 2.4. Mosquito Incubation and Virus Quantification

A subsample of 5 to 9 *Sa. cyaneus* was collected on 3, 4, 5, 7, and 14 dpf from the batch of mosquitoes fed on the mice at 1 dpi and 3, 4, 5, 7, 14, and 21 dpf from the batch of mosquitoes fed on the mice at 2 dpi. A subsample of 10 *Ae. aegypti* was collected on 14 and 21 dpf from each of the batches of mosquitoes fed on mice 1 and 2 dpi. To collect saliva, individual mosquitoes were cold-anesthetized and legs were removed and retained in a microfuge tube containing a steel ball bearing and 500 µL of homogenization media, consisting of 500 µL of DMEM (Gibco, Grand Island, NY, USA) supplemented with 2% FBS, 1% P/S (Gibco) and 2.5 µg/mL amphotericin B (Gibco). Mosquitoes were affixed to a glass slide using mineral oil and the proboscis was inserted into a sterile micropipette tip containing 8 µL FBS and allowed to expectorate for 30 min. The expectorate was then transferred into 100 µL of homogenization media. Finally, the mosquito body was placed into a microtube containing 500 µL homogenization media and a steel ball bearing. Bodies and legs were triturated for 5 min at 26 Hz in a TissueLyser II (Qiagen, Venio, The Netherlands) and clarified by centrifugation.

Virus titer was determined in each sample via plaque-forming unit assay on C6/36 cells using anti-ZIKV hyperimmune serum against ZIKV strain MR-766 at a 1:5000 dilution, and peroxidase-labeled goat anti-mouse secondary antibody (Kirkegaard and Perry Laboratories, Inc., Gaithersburg, MD, USA) at a 1:1000 dilution following previously described methods [15]. The units of resulting titers are given as log_10_ pfu/sample per Hanley et al. [16]. All statistical analysis was conducted using JMP Pro 13 (SAS Institute Inc., Cary, NC, USA).

## 3. Results

Feeding rates for *Sa. cyaneus* were low, with 21% feeding on the mouse at 1 dpi and 28% feeding on the mouse at 2 dpi; in contrast, more than 85% of *Ae. aegypti* fed on the mice on each day. Of 69 engorged *Sa. cyaneus,* ZIKV was detected in only one individual (Table 1), albeit in all body compartments sampled (body, legs, and saliva). This mosquito had fed on a mouse at day 2 dpi; titers increased in the mice by approximately tenfold between day 1 and day 2 pi (5.38 log_10_ pfu/mL to 6.83 log_10_ pfu/mL). Moreover the one infected *Sa. cyaneus* had been allowed to incubate for 21 days, the maximum time period utilized in this study. The small sample size of infected *Sa. cyaneus* precluded statistical analysis of the effects of the experimental manipulations (mouse dpi or mosquito dpf) on infection or a statistical comparison with *Ae. aegypti*.

In contrast, *Ae. aegypti* showed high levels of ZIKV infection, dissemination, and transmission (Table 2). Among mosquitoes fed on the mouse at day 2 pi, 100% showed infection and dissemination by day 14 and 70% showed potential transmission. A nominal logistic analysis was used to test the effects of mouse dpi, mosquito dpf, and their interaction on the percent *Ae. aegypti* infected, disseminated or transmitting. The interaction between mouse dpi and mosquito dpf, as well as mosquito dpf independently, did not have a significant effect on any of these outcomes (*p* > 0.05 for all comparisons). Mouse dpi, which reflects virus titer, did have a significant effect on percent mosquitoes infected (DF = 1, chi squared = 13.68, *p* = 0.0002), the percent mosquitoes in which ZIKV disseminated (DF = 1, chi squared = 21.02, *p* < 0.0001), and the percent of mosquitoes with potential transmission from saliva (DF = 1, chi squared = 13.68, *p* = 0.0002), with higher percentages of ZIKV positive bodies, legs, and saliva among mosquitoes fed on the mouse at 2 dpi.

The titer of ZIKV in the body and legs of the single infected *Sa. cyaneus* fell squarely within the range of ZIKV titers in the bodies and legs of infected *Ae. aegypti* (Table 3). However ZIKV titer in the saliva of *Sa. cyaneus* was almost two orders of magnitude lower than the ZIKV titer in the saliva of *Ae. aegypti* (Table 3).

## 4. Discussion

Here we have shown for the first time that *Sa. cyaneus* is a competent vector for a New World strain of ZIKV, although its competence is orders of magnitude lower than that of *Ae. aegypti*. The minimum extrinsic incubation period (EIP) of ZIKV in *Sa. cyaneus* measured in this study was between 15 and 21 days, substantially longer than the EIP of ZIKV in *Ae. aegypti* measured in this and other studies [2,14]. Although it must be considered preliminary due to the low number of *Sa. cyaneus* infected, this estimate of EIP is consistent with the long EIP of YFV in forest-living New World mosquitoes. Studies conducted in the 1930s and 1940s have shown that the minimum EIP required for various *Haemogogus* species to transmit YFV to monkeys was 13 days, much longer than for *Ae. aegypti* [17,18]. These same studies also showed that the number of mosquitoes capable of transmission increased dramatically between 13 and 21 days post-feeding. Similarly, Couto-Lima et al. [19] reported a minimum YFV EIP of 14 days in *H. leucolaenas* and *S. albiprivus*. Galindo et al. [20] found that the EIP of YFV in *H. mesodentatus gorgasi*, *H. mesodentatus mesodentatus*, *H. equinus* and *S. chloropterus* was ≤26–27 days, but 26 days was the earliest time post-feeding tested. Our data are also in line with studies of the competence of sylvatic *Aedes* species in Africa for ZIKV. Diagne et al. [21] fed four *Aedes* species on artificial bloodmeals containing six strains of ZIKV and monitored virus presence in the body, legs, and saliva at 5, 10, and 15 dpf. They found that only two species, *Ae. (Fredwardsius) vittatus* and *Ae. (Stegomyia) luteocephalus*, expectorated detectable virus, and this occurred only at 15 dpf.

Several caveats to the current study must be acknowledged. First, the *Sa. cyaneus* utilized have been maintained in a colony for decades, whereas the *Ae. aegypti* were only seven generations removed from wild type. Maintenance in a colony can impact vector competence for flaviviruses [22]. However, studies that utilized field collected *Haemagogus* nonetheless showed a long EIP for YFV [17,18,19], suggesting that our observations may reflect the actual dynamics of ZIKV in *Sa. cyaneus*. We have efforts ongoing to collect *Sabethes* and *Haemagogus* mosquitoes in the forest canopies in and around Manaus, Brazil in order to establish collections of field-caught and low-generation colony mosquitoes for vector competence testing. Second, a relatively small number of *Sa. cyaneus* fed on infected mice. This species is extremely difficult to maintain in colony and does not readily feed on mice; even under our tailored feeding protocol, a maximum 28% feeding efficiency was observed. Third, we utilized only one strain of ZIKV. We and others have previously shown that ZIKV strains differ in their ability to infect, disseminate and be transmitted in saliva in *Ae. aegypti* [14,23] and *Ae. (Stegomyia) albopictus* [24], and it therefore seems likely that strains may also vary in their ability to infect *Sa. cyaneus*. Nonetheless, we do know that the ZIKV strain used in the current study is quite infectious for both *Ae. aegypti* (this study) and *Ae. albopictus* [24]. Finally, the ZIKV titers achieved in A129 mice were several orders of magnitude higher than those reported for humans, i.e., [25,26,27] or New World non-human primates [10,11,12], although these comparisons are indirect as most of the human and non-human primate studies quantified viremia via measurement of the viral genome rather than infectious virus. Generally, the percentage of mosquitoes infected is positively correlated with arbovirus titer in the bloodmeal [28].

Despite these limitations, the current study offers the first view of vector competence and EIP of any New World sylvatic mosquito vector for ZIKV. EIP is a critical parameter for models of arbovirus transmission [29], Although we were not able to measure the EIP_50_ of ZIKV in *Sa. cyaneus* due it its low overall competence [30], nonetheless, the data generated here can be used to refine models of ZIKV spillback [6]. While *Sabethes cyaneus* has not itself been implicated as a vector of any arbovirus, other *Sabethes* species have been shown to carry the flaviviruses YFV [31,32,33,34,35], dengue virus [36], and Ilheus virus [37] as well as the rhabdovirus Xiburema virus [38]. *Sabethes* species are highly abundant in the forest canopy [39,40,41,42], occur in forested areas of large cities [43,44,45], have long lifespans in nature that exceed the 21 day EIP measured here [46], and have been collected via human land catch [47,48]. Together, these traits suggest that species in this genus have high potential act as bridge vectors [49] for spillback from humans to wildlife.

## Figures and Tables

**Table 1:** Infection, dissemination, and potential transmission of ZIKV in *Sabethes cyaneus* fed on infected A129 mice.

Mosquito Day Post-Feeding	Mouse Day Post-Infection	ZIKV Titer (log_10_ pfu/mL)	N	No. (%) Infection	No. (%) Dissemination	No. (%) Transmission
3	1	5.38	5	0	0	0
4	1	5.38	5	0	0	0
5	1	5.38	5	0	0	0
7	1	5.38	5	0	0	0
14	1	5.38	5	0	0	0
3	2	6.83	7	0	0	0
4	2	6.83	7	0	0	0
5	2	6.83	7	0	0	0
7	2	6.83	7	0	0	0
14	2	6.83	7	0	0	0
21	2	6.83	9	1 (11.1)	1 (11.1)	1 (11.1)

**Table 2:** Infection, dissemination, and transmission of ZIKV in *Aedes aegypti*.

Mosquito Day Post-Feeding	Mouse Day Post-Infection	ZIKV Titer (log_10_ pfu/mL)	N	No. (%) Infection	No. (%) Dissemination	No. (%) Transmission
14	1	5.38	10	3 (30)	1 (10)	0 (0)
21	1	5.38	10	4 (40)	2 (20)	2 (20)
14	2	6.83	10	10 (100)	10 (100)	7 (70)
21	2	6.83	10	10 (100)	10 (100)	7 (70)

**Table 3:** ZIKV titer in infected tissues from *Sabethes cyaneus* and *Ae. aegypti*.

Species	Mosquito Day Post Feeding	Mouse Day Post Infection	Mean Body Titer * (log_10_ pfu/mL) ± 1SE (N)	Mean Legs Titer * (log_10_ pfu/mL) ± 1SE (N)	Mean Saliva Titer * (log_10_ pfu/mL) ± 1SE (N)
*Sabethes cyaneus*	21	2	5.4 (1)	4.4 (1)	1.1 (1)
*Aedes aegypti*	14	1	4.5 ± 0.2 (3)	3.2 (1)	NA (0)
	21	1	5.1 ± 0.2 (4)	4.4 ± 0.4 (2)	2.8 ± 1.0 (2)
	14	2	5.2 ± 0.2 (10)	4.4 ± 0.4 (10)	2.8 ± 0.3 (7)
	21	2	5.7 ± 0.1 (10)	4.7 ± 0.3 (10)	2.9 ± 0.5 (7)

* Mean values are calculated from samples with detectable virus.

## Supplementary Materials

Supplementary materials can be found at http://www.mdpi.com/1999-4915/10/8/434/s1.
